# Congenital cystic adenomatoid malformations of the lung: an epithelial transcriptomic approach

**DOI:** 10.1186/s12931-020-1306-5

**Published:** 2020-02-04

**Authors:** Guillaume Lezmi, Shamila Vibhushan, Claudia Bevilaqua, Nicolas Crapart, Nicolas Cagnard, Naziha Khen-Dunlop, Christine Boyle-Freyssaut, Alice Hadchouel, Christophe Delacourt

**Affiliations:** 1 Service de Pneumologie et d’Allergologie Pédiatriques, AP-HP, Hôpital Universitaire Necker-Enfants Malades, 75743 Cedex 15 Paris, France; 2 INSERM, U955, Institut Mondor de Recherche Biomedicale (IMRB), Equipe 4, 94000 Créteil, France; 3 Paris Descartes University, Paris, France; 4 Institut National de la Recherche Agronomique (INRA), AgroParisTech, Université Paris-Saclay, Jouy en Josas, France; 5 Inserm UMR1163, Imagine Institute, Genomics Core Facility, Paris, France; 6 Service de Chirurgie Pédiatrique, AP-HP, Hôpital Universitaire Necker-Enfants Malades, 75743 Cedex 15 Paris, France

**Keywords:** Congenital thoracic malformations, Cystic lung, Transcriptome, Transforming growth factor, Laser capture microdissection

## Abstract

**Background:**

The pathophysiology of congenital cystic adenomatoid malformations (CCAM) of the lung remains poorly understood.

**Aim:**

This study aimed to identify more precisely the molecular mechanisms limited to a compartment of lung tissue, through a transcriptomic analysis of the epithelium of macrocystic forms.

**Methods:**

Tissue fragments displaying CCAM were obtained during planned surgical resections. Epithelial mRNA was obtained from cystic and normal areas after laser capture microdissection (LCM). Transcriptomic analyses were performed and the results were confirmed by RT-PCR and immunohistochemistry in independent samples.

**Results:**

After controlling for RNA quality, we analysed the transcriptomes of six cystic areas and five control areas. In total, 393 transcripts were differentially expressed in the epithelium, between CCAM and control areas. The most highly redundant genes involved in biological functions and signalling pathways differentially expressed between CCAM and control epithelium included *TGFB2, TGFBR1*, and *MAP 2 K1*. These genes were considered particularly relevant as they have been implicated in branching morphogenesis. RT-qPCR analysis confirmed in independent samples that *TGFBR1* was more strongly expressed in CCAM than in control tissues (*p* < 0.03). Immunohistochemistry analysis showed TGFBR1 (*p* = 0.0007) and TGFB2 (*p* < 0.02) levels to be significantly higher in the epithelium of CCAM than in that of control tissues.

**Conclusions:**

This compartmentalised transcriptomic analysis of the epithelium of macrocystic lung malformations identified a dysregulation of TGFB signalling at the mRNA and protein levels, suggesting a possible role of this pathway in CCAM pathogenesis.

**Trial registration:**

ClinicalTrials.gov Identifier: NCT01732185.

## Background

Congenital cystic adenomatoid malformations (CCAM) of the lung are the most frequent congenital thoracic malformations [1]. Their pathophysiology remains poorly understood. The sporadic occurrence of CCAM, their focal location, the normality of the lung surrounding the lesion, the absence of associated congenital abnormalities, and the expression of markers of lung development within the lesions suggest that CCAM result from a transient, focal, non-hereditary disruption of normal lung development [2, 3]. Our current understanding of the pathogenesis of CCAM is based largely on a candidate molecule hypothesis derived from animal models. Airway morphogenesis is thought to be driven by complex interactions between the epithelium and the underlying mesenchyme mediated by diffusible growth factors, such as fibroblast growth factor 10 (FGF-10) and sonic hedgehog (SHH) [4, 5]. Several experimental studies have implicated the SHH-FGF10 axis in the pathogenesis of cystic abnormalities during lung development [3]. FGF10 and its receptor, FGFR2b, are strongly expressed in the mesenchyme and epithelium of human CCAM, differentiating these abnormalities from other cystic abnormalities, such as type 1 pleuropneumoblastoma [6]. However, the levels of expression of these molecules do not differ between CCAM and control tissues [6]. A recent analysis of the human congenital lung lesion transcriptome revealed an upregulation of epithelium-related genes, and a dysregulation of the expression of genes related to the Ras and PI3K-AKT-mTOR signalling pathways [7]. Neither FGF10 nor FGFR2b was found to be dysregulated. However, the use of whole lungs in this study may have biased gene identification against genes with expression limited to a particular compartment of the lung parenchyma. Furthermore, only two macrocystic lesion transcriptomes were analysed. These lesions appeared to cluster separately from microcystic and hybrid lesions, and were excluded from the analysis. It this study, we performed a transcriptomic analysis of the epithelium of macrocystic forms. The tissues were isolated by laser capture microdissection, and the transcriptomes of the epithelial cells in cystic zones and adjacent healthy tissue areas were analysed. Our results suggested a potential role for TGF-beta signalling. We validated these results by PCR and immunohistochemistry, which confirmed the differences in expression of these molecules between zones of malformation and healthy tissue.

## Materials and methods

This study was approved by the institutional ethics committee (*Comité de Protection des Personnes-Ile de France VII* (N°12–025)), and by the French national drug safety agency, the *Agence Nationale de Sécurité du Médicament* (B120601–10). Written informed consent was obtained from the parents of the patients.

### Patient selection

Children with congenital cystic pulmonary malformation were recruited during follow-up visits at the Paediatric Pulmonology Department of Necker Hospital. Tissue fragments were collected prospectively during programmed surgical removal of the malformation, usually at about 6 months of age. The diagnosis of CCAM was confirmed after surgical resection, by histological analysis, and CCAM were classified according to the Stocker classification [8]. Non-cystic pulmonary malformations or hybrid lesions were excluded from the study. Children who had an infection of their malformation before surgery were also excluded. Infection was defined by fever and a new radiological infiltrate at the location of the malformation.

### Tissue acquisition

After resection, the explant was immediately cooled for a few seconds in PBS buffer at 4 °C to reduce the activity of endogenous RNAases. Several fragments were rapidly collected on ice, placed in cryomolds and cryotubes and immersed in liquid nitrogen for storage at − 80 °C. These fragments were used for laser microdissection and RT-qPCR analysis. For each CCAM, we collected tissues from two areas, defined on the basis of their macroscopic appearance: a cystic area and an area of normal appearance. The macroscopically normal tissue was collected from the area surrounding the lesion, and was used as a control if this normal appearance was also confirmed microscopically (see below). The remainder of the explant was used for histological analysis by a pathologist to confirm the diagnosis of CCAM.

### Laser capture microdissection

**Fig. 1:**
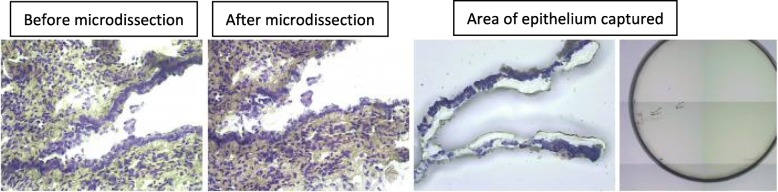
Laser capture microdissection of epithelial samples

We cut 8 μm-thick sections, which were then stained with cresyl violet for 30 s, rinsed in 95% ethanol and dehydrated in 100% ethanol. The sections were immersed in xylene to eliminate the ethanol, dried in room air and placed in a vacuum chamber until use. The sections were then microdissected with a VERITAS laser dissecting microscope (Arcturus, Life Technologies) at the ICE core facility (INRA, Jouy-en-Josas). Slides were viewed at × 20 or × 40 magnification, and the epithelium was delineated on both the cystic and control slides. Normal and abnormal structures are difficult to differentiate in CCAM. This first step was therefore also used to confirm that the macroscopically normal tissues did not contain any abnormal structures. The epithelium tissues from cystic and normal areas were then dissected out and collected (Fig. 1). They were immediately placed in RNA extraction buffer (Arcturus Picopure RNA isolation kit) and stored at − 80 °C until extraction. Total RNA was isolated with the Arcturus Picopure kit (Life Technologies), according to the manufacturer’s instructions.

### Assessment of the quality of the extracted RNA

RNA quality was assessed with an Agilent Model 2100 Bioanalyser (Agilent Technologies). An RNA integrity number (RIN) greater than 7 was considered acceptable for the analysis. The quality of the RNA extracted from the first cut (control block) was used to assess the quality of the RNA in the work piece. The RIN of the RNA extracted from the control blocks ranged from 7.5 to 8.5, reflecting partial degradation of the RNA during the surgical procedure. As recommended [9], Agilent analysis was also systematically performed on the RNA obtained from the tissue remaining on the slides after microdissection (control slide), to assess the degradation of the RNA by the procedure. For small amounts of extracted RNA (< 150 μg/μl), the RIN obtained was artificially low. In such cases, if the RIN on the corresponding control slide exceeded 7, the sample was considered to be of sufficient quality for further analysis.

There is no broad consensus RIN threshold for the inclusion of samples in transcriptomic analyses, but RIN scores higher than 6 were required for the Genotype-Tissue Expression (GTEx) project (https://gtexportal.org/home/documentationPage).

### Transcriptomic analysis

An expression analysis was performed with the GeneChip® Human Exon 1.0 ST Array (Affymetrix), investigated more than one million exon clusters within the known and predicted transcribed regions of the entire genome (the genomics core facility of the Imagine Institute, Paris). Two kits from NuGEN (NuGEN Technologies) were used for the amplification and post-amplification treatment of samples before hybridisation. Using 200 pg of total RNA as the starting material, we subjected samples to Ribo-SPIA RNA Amplification (SPIA, for single primer isothermal amplification). The amplicons obtained were fragmented and labelled with biotin, with the One-Direct System and the Encore Biotin Module, as recommended by the manufacturer. The biotinylated DNA fragments obtained were hybridised with the Human Exon 1.0 ST Array, with the Hybridization Wash Stain kit (Affymetrix). The arrays were washed, stained, and scanned with an Affymetrix Model 450 Fluidics Station, an Affymetrix Model 3000 scanner and Command Console software, which piloted the GeneChip systems.

Fluorescence data were imported into two software suites for analysis: Affymetrix® Expression Console™ and R Bioconductor. Gene expression was evaluated by calculating the RMA algorithm in Expression Console, and flags were computed with a custom algorithm in R. Assuming that no more than 80% of genes are expressed, we selected the lowest 20% of values obtained for each microarray for use as the background. The threshold was fixed at two standard deviations above the mean of the background values. All probes with normalised intensity values below this threshold were flagged as 0 rather than 1. Lists were created by filtering for probes flagged as “Present” (flag = 1) for at least half the chips. Groups were compared in Student’s *t* tests. False discovery rates were estimated by filtering the resulting *p* values at 5%. Hierarchical clustering was performed with Spearman’s rho valus and the average link algorithm. Data were subjected to Ingenuity Pathway Analysis (IPA) for the modelling of networks, to identify the relevant pathways and biological processes. The filters *p* value ≤0.05 and a 1.2-fold change in probe expression level were used to identify transcripts differentially expressed in the epithelium, between CCAM and control areas. Gene-set enrichment analysis was performed with Ingenuity software.

### RT-qPCR analysis

For confirmation of the results of transcriptomic analysis, we used RT-PCR on small amounts of preamplified cDNA (QuantiTect® Whole Transcriptome, Qiagen) to assess gene expression levels. We used the following primers:

**TGFb2** (NM_003238.3) F: ATGCCAACTTCTGTGCTGGA R: AAATCTTGGGACACGCAGCA, **TGFbR1** (NM_004612,3) F: CTCCAAACCACAGAGTGGGAA R: ATCATCGAGAACTTCAGGGGC, **MAP 2 K1** (NM_002755,3) F: CGCCCATCCAGCTGAACC, R: TCATCAAGCTCTAGCTCCTCCA.

### Immunohistochemistry

After resection, the tissues were embedded in paraffin and 5 μm sections were cut for immunohistochemistry. Sections were processed as previously described [6], and incubated with the primary antibodies for TGFb2 (1:200, PA5–32629, Thermo Fisher Scientific Inc., Waltham, MA), TGFbR1 (abcam, ab31013, 1:200); MEK1/MAP 2 K1, Thermo Fisher Scientific, MA1–095, 1:10000) and with isotype control rabbit IgG (02–6102, Life Technologies, Carlsbad, USA) or mouse IgG, (Dako, X0931), with 1.5% normal horse serum (PK-7800, RTU Vectastain, Vector Laboratories, Burlingame, USA) in PBS. For each patient, and for each immunostaining reaction, we photographed 10 zones of epithelium selected at random, at a magnification of × 20. Analyses were performed with Image J analysis software (http://rsb.info.nih.gov/ij/). Immunostaining in epithelial cells (brown) and nucleus counterstaining (purple) signals were separated and converted into greyscale intensities with the Colour Deconvolution plugin of Image J software. Two of the authors (GL and SV) analysed the staining blind and independently of each other. The ratio of immunostaining to counterstaining intensities was calculated for each Image.

### Analysis

The values obtained in RT-PCR and immunostaining analyses were compared in Mann-Whitney tests, with *p* values < 0.05 considered significant.

## Results

### Transcriptomic analysis

Following LCM, RNA of sufficient quality was isolated from 10 patients (Additional file 1: Table S1). For one patient, mRNA was obtained from both cystic and control areas. For five patients, mRNA was obtained exclusively for the cystic area, and, for another four patients, mRNA was obtained only from the control area. In total, we analysed six cystic areas and five control areas. The median age of the six patients for whom cystic areas (3 type I, 3 type II CCAM) were analysed was 5.7 months (range: 4.2–7.8 months), and did not differ from that of the patients in whom control areas were analysed (8 months (range: 4.2–13.8 months)), *p* = 0.88.

**Fig. 2:**
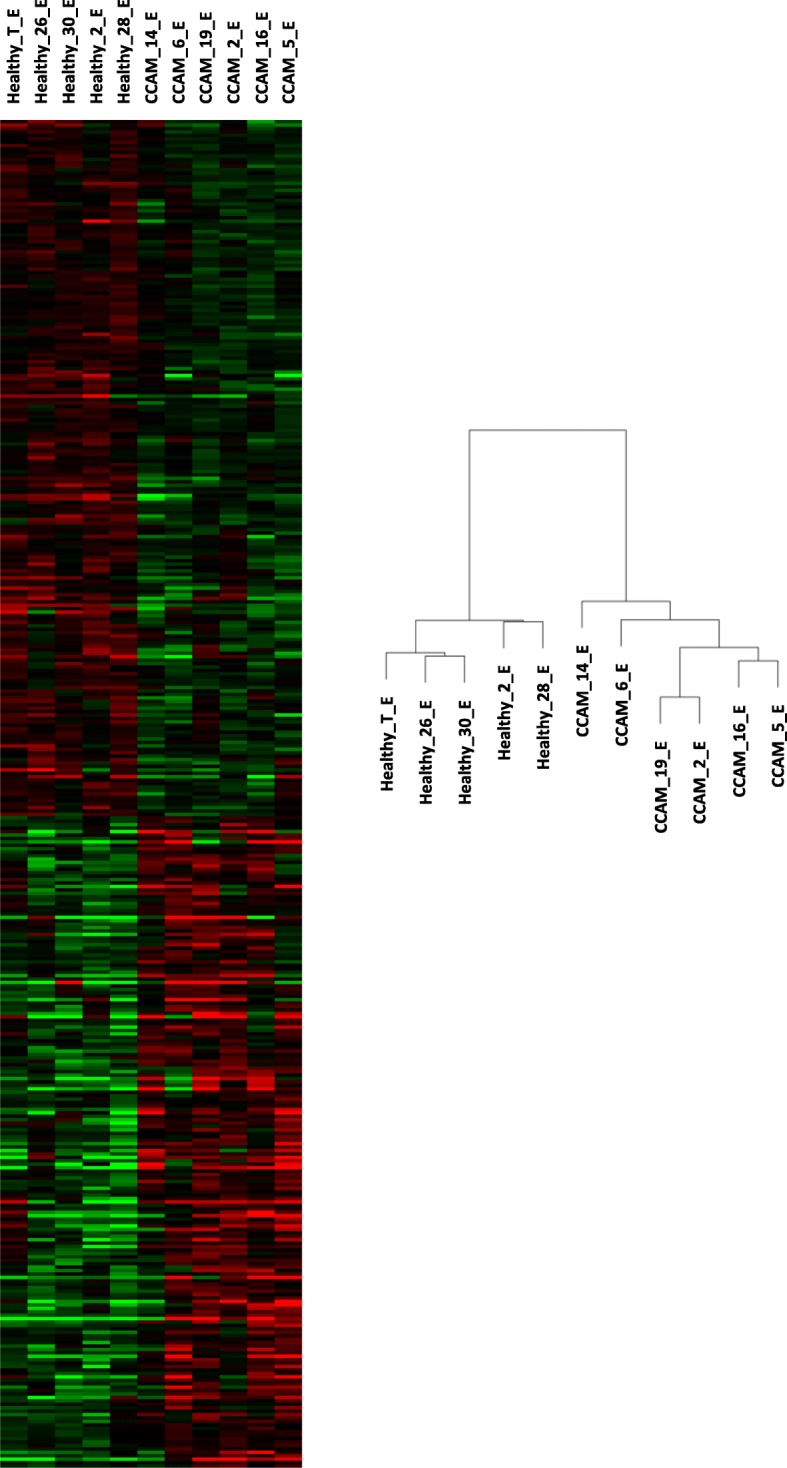
Unbiased clustering of genes based on their levels of expression in microarray analyses of epithelial samples from six cystic and five control areas. Samples CCAM_2_E, CCAM_6_E and CCAM_19_E are type I CCAM; samples CCAM_5_E, CCAM_14_E and CCAM_16_E are type II CCAM. The cluster correlation dendrogram showed a clear separation of CCAM samples from control samples. No clear clustering differentiated type I CCAM from type II CCAM

**Fig. 3:**
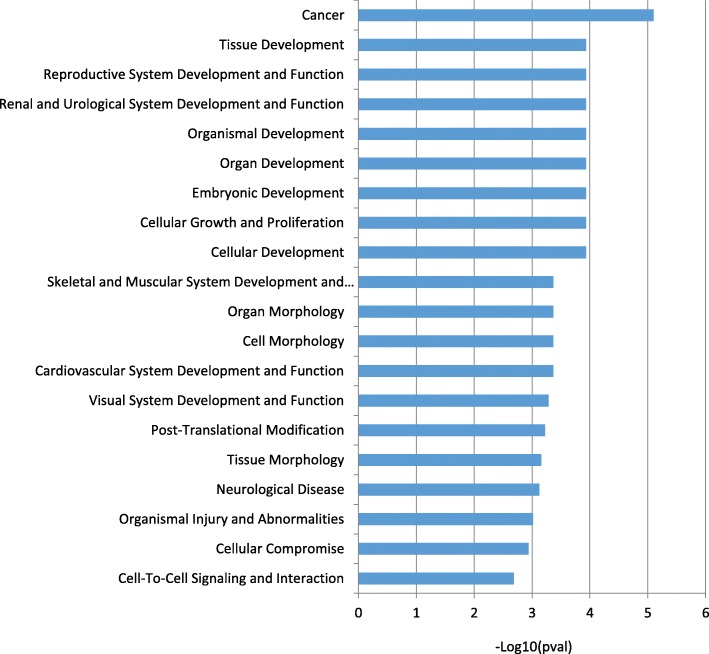
Gene ontology analysis of genes differentially expressed between CCAM epithelium and control epithelium. *TGFB2* was identified in 17 of the top 20 biological processes, and TGFbR1 in 12 of the top 20 biological processes

**Fig. 4:**
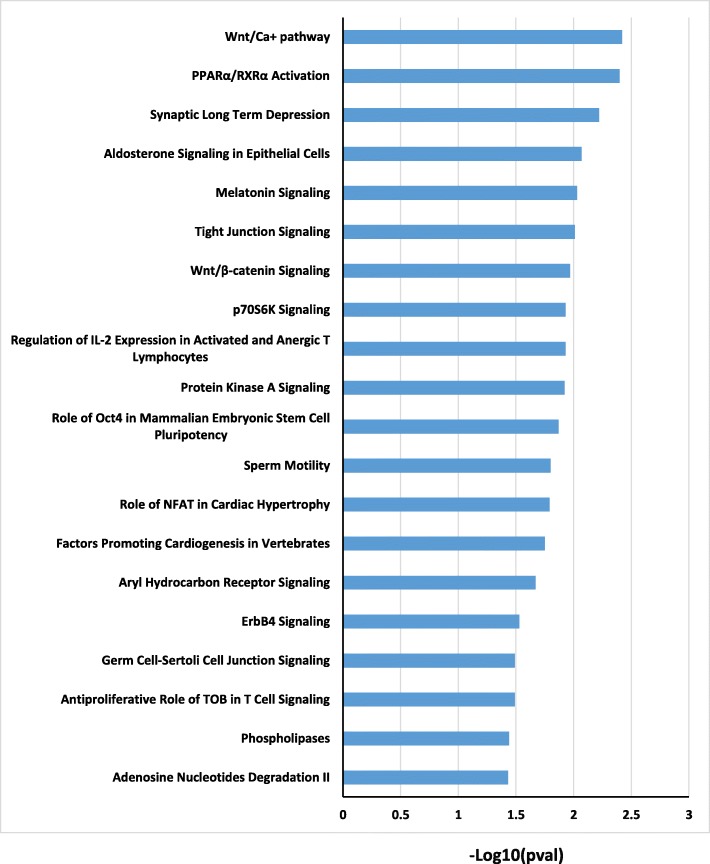
Gene-set enrichment analysis of genes differentially expressed between cystic and control areas of epithelium. TGFB2 was identified in 10 of the top 20 signalling pathways, and TGFbR1 in 9 of the top 20 signalling pathways

We observed marked differences in the global profile of epithelial transcripts between CCAM and control areas (Fig. 2). Using Ingenuity software with filtering on a *p* value ≤0.05 and a 1.2-fold difference in probe expression, we identified 393 genes as differentially expressed in the epithelium, between CCAM and control areas: 203 were downregulated (Additional file 1: Table S2), and 190 upregulated (Additional file 1: Table S3). Gene ontology analysis of the genes differentially expressed between CCAM epithelium and control epithelium showed that several of the significantly dysregulated genes were related to cancer and developmental processes (Fig. 3). Gene-set enrichment analysis identified groups of genes that were significantly enriched or depleted as belonging to signalling pathways (Fig. 4, Additional file 1: Table S4). To select candidate genes for confirmation by qPCR and immunohistochemistry, we considered redundant genes within the 20 signalling pathways differing most significantly between CCAM and controls (Fig. 4). Ninety seven genes were identified within these 20 signalling pathways (Additional file 1: Table S4). Only 6 genes were highly redundant: PLCD4 (11 pathways), PRKCA (11 pathways), MAP 2 K1 (10 pathways), PLCE1 (10 pathways), TGB2 (10 pathways), and TGFBR1 (9 pathways). We selected *TGFB2, TGFBR1*, and *MAP 2 K1* for further analysis, because these genes had already been implicated in branching morphogenesis [10, 11]. Predicted upstream pathway analysis identified genes involved in lung development, such as *FGF1, WNT11, CEBPA, CREB1*, and *HIF1A*, as potential upstream regulators of groups of genes including *TGFB2, TGFBR1* or *MAP 2 K1* (Additional file 1: Table S5).

### Confirmation of the dysregulated expression of candidate genes in independent samples

**Fig. 5:**
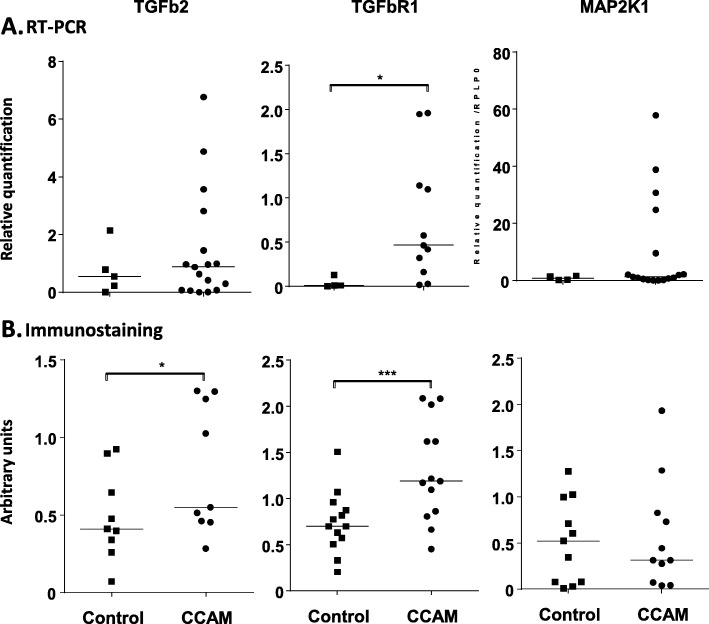
RT-PCR (**a**) and immunostaining (**b**) results for TGFb2, TGFbR1 and MAP 2 K1 in control and CCAM samples. TGFB2: 5 controls vs. 17 CCAM samples and 9 controls vs. 9 CCAM samples for RT-PCR and immunostaining, respectively; TGFBR1: 4 controls vs. 11 CCAM samples and 13 controls vs. 13 CCAM samples for RT-PCR and immunostaining, respectively; MAP 2 K1: 4 controls vs. 17 CCAM samples and 13 controls vs. 11 CCAM samples for RT-PCR and immunostaining, respectively. * *p* < 0.05; ***p < 0.001

We then analysed the levels of mRNA and protein for TGFB2, TGFBR1 and MAP 2 K1 in tissue samples from 17 new patients. The RT-qPCR data for each gene were normalised against the levels of 18S rRNA, beta 2 microglobulin and RPLP0. TGFBR1 mRNA levels were significantly higher in CCAM than in control tissues (*p* < 0.03), whereas no significant difference was found in the levels of mRNA for TGFB2 or MAPK21 (Fig. 5a).

**Fig. 6:**
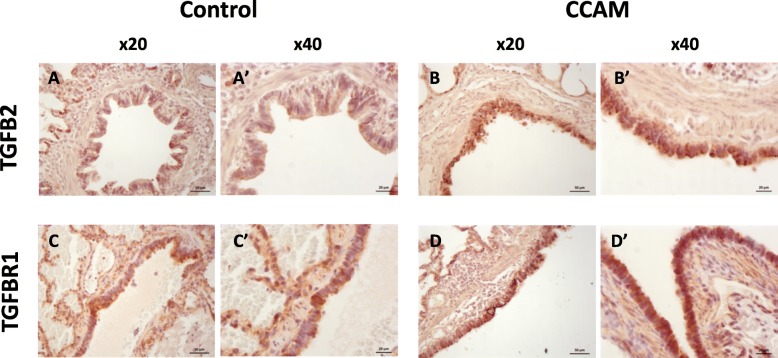
Immunostaining for TGFB2 (A, A', B, B') and TGFBR1 (C, C', D, D') in CCAM (B, B', D, D') and control samples (A, A', C, C') , at magnifications of × 20 (A, C, B, D) and × 40 (A', B', C', D'). Diffuse expression is observed in CCAM epithelium

Immunohistochemistry showed the levels of TGFBR1 (*p* = 0.0007) and TGFB2 (*p* < 0.02) in the epithelium to be significantly higher in CCAM than in control tissues. No difference in MAP 2 K1 protein levels was observed between CCAM and control tissues (Fig. 5b). TGFB2 and TGFBR1 were expressed in a diffuse manner throughout the epithelium of CCAM tissues, but were expressed only sparsely in the control epithelium (Fig. 6). No expression of TGFB2 was observed in the mesenchyme. A low expression of TGFBR1 was observed in the mesenchyme, with no difference between controls and CCAM.

## Discussion

We used a laser microdissection-based protocol to separate out CCAM and control areas of the epithelium. We then demonstrated differences in the gene expression profiles of the CCAM and control tissues. *TGFB2* and *TGFBR1* were among the genes identified as belonging to the epithelial biological processes and signalling pathways differing most significantly between CCAM and control tissues. The overexpression of these genes was confirmed in analyses of CCAM and control samples from additional patients. This study therefore provides the first evidence for a role of TGF in the pathophysiology of congenital macrocystic lung malformations.

During lung development, the epithelium and surrounding mesenchyme display specific patterns of gene expression [12]. Analyses on whole-tissue homogenates may therefore be inconclusive or misleading, as the contribution of a specific tissue compartment may be masked. In addition, the term “CCAM” covers a number of different malformation phenotypes, including microcystic and macrocystic forms, with or without systemic vascularisation, and it remains unknown whether these different phenotypes share the same molecular dysfunctions. We aimed to homogenise our analysis as much as possible, by focusing on a single compartment (the epithelium) in a single malformation phenotype (macrocystic forms). LCM was used for the epithelium-specific molecular profiling of areas of malformation and adjacent healthy zones. The LCM procedure itself and the analysis of microdissected material can be challenging, particularly due to the small amounts of nucleic acids isolated and the loss of RNA due to degradation during the process. With the most recent guidelines for LCM, we were able to obtain sufficient amounts of RNA with limited degradation [9, 13].

Our results indicate significant differences in gene expression between the epithelia of CCAM and healthy tissues. Dysregulated lung epithelium was recently suggested to play a key role in branching morphogenesis in congenital lung malformations, in a study on whole lung tissues corresponding to different malformation phenotypes [7]. Our findings confirm that epithelial dysfunction makes a significant contribution to the pathogenesis of pulmonary malformations. Furthermore, our findings identify TGB2 and TGFBR1 as key molecules in epithelial pathway dysregulation, and confirm their overexpression in additional samples. These findings are consistent with a causal role for TGFβ signalling in CCAM. The TGFβ signalling pathway is involved in various biological processes, including development and extracellular matrix production, and is crucial for epithelial-mesenchymal interactions during lung branching morphogenesis and alveolarisation [11, 14]. A dimeric form of activated TGFβ binds to the transmembrane TGFBR1 and TGFBR2 receptors [11]. TGFBR2 phosphorylates TGFBR1, which activates the canonical Smad-dependent pathway [11, 15]. During airway morphogenesis, different expression patterns have been described for the different isoforms of TGFB, TGFB2 being localised in the distal epithelium, whereas TGFB1 and TGFBR3 are expressed in the mesenchyme [16]. Several experimental studies have shown TGF signalling to be crucial for lung branching [11]. Branching morphogenesis is disrupted by lung mesenchymal knockouts of the TGFB1 gene [17]. More specifically, TGFB2-knockout mice develop a number of specific developmental defects, including collapsed distal airways [18]. The epithelium-specific deletion of *TGFBR1* in mouse lungs leads to an enlargement of the airways, with disorganisation of the epithelium [19]. In an organ culture model, TGFB2 was found to inhibit lung explant branching 50 times more potently than TGFB1 [20]. Based on these experimental data, our results suggest that the dysregulation of TGF signalling in the pulmonary epithelium plays a direct role in CCAM pathogenesis. However, one limitation of our study, as in all human studies on these malformations, was the postnatal collection of the tissues, at a time point well after the triggering molecular event in the pseudoglandular phase of development. The observed signalling anomalies may, therefore, be no more than a consequence of the initial molecular dysregulation, rather than the actual triggering event.

The hypothesis of a direct role for TGF signalling in the pathogenesis of CCAM is also supported by our upstream transcriptomic study, which identified several developmental genes as potential upstream regulators of groups of genes including *TGFB2* or *TGFBR1* already shown to be involved in the branching process. CEBPA is crucial for the proximo-distal differentiation of the pulmonary epithelium [21]. The relationship between TGFβ signalling and CEBPA-mediated transcriptional regulation has already been demonstrated during liver development [22]. HIF1A plays a major role in early pulmonary vasculogenesis, and its signalling is amplified through interactions between the mammalian target of rapamycin (mTOR) andFGF-10 signaling [23], both of which are dependent on TGF for activation [17, 24]. WNT11 has been shown to interact directly with TGF signalling to induce the production of smooth muscle alpha-actin [25]. FGF1 is expressed very early in the mouse lung epithelium, during branching, and has been shown to induce branching morphogenesis in Matrigel-embedded E11 epithelium [26]. FGF1 has also been shown to interact with TGF-driven fibroblast-to-myofibroblast transdifferentiation [27].

In conclusion, this transcriptomic analysis of the epithelium of CCAM tissues reveals a dysregulation of TGFB signalling at the mRNA and protein levels. These data are consistent with previous studies demonstrating the involvement of TGFB signalling in airway morphogenesis, and thus identify a possible role for this signalling pathway in CCAM pathogenesis.

## Supplementary information

**Additional file 1: Table S1.** RIN and mRNA concentrations from samples used for transcriptome analysis. **Table S2.** List of downregulated genes in CCAM epithelium compared to control zones. These genes were selected using Ingenuity software with a filter *p* value ≤0.05 and a 1.2-fold alteration of probe expression. **Table S3.** List of upregulated genes in CCAM epithelium compared to control zones. These genes were selected using Ingenuity software with a filter *p* value ≤0.05 and a 1.2-fold alteration of probe expression. **Table S4.** Pathway analysis. **Table S5.** Upstream analysis.

## Data Availability

The datasets used and/or analysed during the current study are available from the corresponding author on reasonable request.

## Abbreviations

CCAM: Congenital cystic adenomatoid malformation
FGF: Fibroblast Growth Factor
LCM: Laser capture microdissection
SHH: Sonic hedgehog
TGF: Transforming Growth Factor
