# Supplementary material for: Congenital cystic adenomatoid malformations of the lung: an epithelial transcriptomic approach
Source: Respir Res. 2020 Feb 4;21:43. doi: 10.1186/s12931-020-1306-5 (PMC7001206; doi:10.1186/s12931-020-1306-5)
Supplement: Supplementary file 1 — Additional file 1: Table S1. RIN and mRNA concentrations from samples used for transcriptome analysis. Table S2. List of downregulated genes in CCAM epithelium compared to control zones. These genes were selected using Ingenuity software with a filter p value ≤0.05 and a 1.2-fold alteration of probe expression. Table S3. List of upregulated genes in CCAM epithelium compared to control zones. These genes were selected using Ingenuity software with a filter p value ≤0.05 and a 1.2-fold alteration of probe expression. Table S4. Pathway analysis. Table S5. Upstream analysis. [file 12931_2020_1306_MOESM1_ESM.docx]

Table S1. RIN and mRNA concentrations from samples used for transcriptome analysis

|  | RIN | pg/µl |
| --- | --- | --- |
| CCAM 1 | 7.5 | 85 |
| CCAM 2 | 7.1 | 1123 |
| CCAM 3 | 7.5 | 1351 |
| CCAM 4 | 6.8 | 282 |
| CCAM 5 | 7.4 | 155 |
| CCAM 6 | 7.4 | 254 |
| Control 1 | 7.6 | 67 |
| Control 2 | 7.2 | 127 |
| Control 3 | 7.2 | 77 |
| Control 4 | 7.2 | 466 |
| Control 5 | 8 | 774 |

Table S2. List of downregulated genes in CCAM epithelium compared to control zones. These genes were selected using Ingenuity software with a filter p value ≤ 0.05 and a 1.2-fold alteration of probe expression.

| **Probes** | **Gene Symbol** | **pval** | **Mean Ratio CCAM vs Controls** |
| --- | --- | --- | --- |
| 2408028 | NT5C1A | 2,33E-04 | 0,809706397 |
| 2319225 | H6PD | 6,14E-04 | 0,791446591 |
| 3203413 | B4GALT1 | 1,21E-03 | 0,640464213 |
| 3157434 | RHPN1-AS1 | 1,21E-03 | 0,782043771 |
| 3638607 | ANPEP | 1,48E-03 | 0,809272656 |
| 2363679 | --- | 1,73E-03 | 0,806682289 |
| 3695359 | FAM96B // FAM96B | 2,30E-03 | 0,436551274 |
| 3608787 | SLCO3A1 | 2,70E-03 | 0,799888426 |
| 3638665 | AP3S2 // AP3S2 | 2,77E-03 | 0,327636284 |
| 3592420 | HMGN2P46 // HMGN2P46 | 3,58E-03 | 0,763301441 |
| 3678542 | C16orf89 | 4,03E-03 | 0,821049349 |
| 2717846 | GPR78 | 4,04E-03 | 0,697985325 |
| 3079369 | TMUB1 | 4,21E-03 | 0,830608966 |
| 2527895 | PLCD4 | 4,24E-03 | 0,831964523 |
| 3137120 | CA8 | 4,31E-03 | 0,629599366 |
| 2565163 | --- | 4,45E-03 | 0,377963782 |
| 2949299 | LY6G6C | 4,54E-03 | 0,813255449 |
| 3131741 | RAB11FIP1 | 4,67E-03 | 0,761023546 |
| 3980964 | --- | 4,85E-03 | 0,723140984 |
| 2900750 | OR2J3 | 5,69E-03 | 0,616327634 |
| 3952703 | C22orf39 | 5,88E-03 | 0,831419418 |
| 2647792 | SELT | 6,02E-03 | 0,644770446 |
| 2796338 | LOC728175 | 6,26E-03 | 0,784720314 |
| 2356100 | HFE2 | 6,60E-03 | 0,807295124 |
| 2948379 | GNL1 | 7,08E-03 | 0,795774995 |
| 3168409 | CCIN | 7,63E-03 | 0,679884016 |
| 3360486 | OR51B4 | 7,96E-03 | 0,755376619 |
| 2886535 | LOC100133106 | 8,38E-03 | 0,795848902 |
| 2639129 | DIRC2 | 8,63E-03 | 0,548459891 |
| 2324097 | PINK1 | 8,72E-03 | 0,772495968 |
| 3216671 | CTSL2 | 8,75E-03 | 0,686686498 |
| 2560149 | CCDC142 // CCDC142 | 8,78E-03 | 0,80560081 |
| 2406253 | --- | 9,16E-03 | 0,501971334 |
| 2565161 | --- | 9,17E-03 | 0,564098987 |
| 3451708 | TWF1 | 9,31E-03 | 0,759376633 |
| 2941757 | ERVFRD-1 | 9,70E-03 | 0,806721274 |
| 3029498 | OR2A2 | 9,96E-03 | 0,752152425 |
| 2458629 | LEFTY2 | 1,02E-02 | 0,777083668 |
| 3955915 | TPST2 | 1,05E-02 | 0,817472847 |
| 2491676 | VAMP5 | 1,08E-02 | 0,552800094 |
| 3514685 | LINC00282 | 1,09E-02 | 0,792426811 |
| 2561955 | SUCLG1 | 1,11E-02 | 0,703120258 |
| 3127610 | PEBP4 | 1,11E-02 | 0,352729176 |
| 2647458 | RNF13 | 1,21E-02 | 0,74771132 |
| 2946369 | HIST1H3G | 1,29E-02 | 0,740881467 |
| 2320411 | AGTRAP | 1,36E-02 | 0,818054157 |
| 3602299 | NEIL1 | 1,37E-02 | 0,807227052 |
| 2880830 | IL17B | 1,40E-02 | 0,751046131 |
| 2849469 | ANKH | 1,40E-02 | 0,803346802 |
| 3079172 | TMEM176B | 1,43E-02 | 0,770754516 |
| 3145973 | --- | 1,45E-02 | 0,744183165 |
| 3374856 | MRPL16 | 1,52E-02 | 0,732479159 |
| 2618702 | ZNF620 | 1,56E-02 | 0,528078509 |
| 2898371 | NRSN1 | 1,63E-02 | 0,784484948 |
| 2883283 | TIMD4 | 1,64E-02 | 0,797819912 |
| 3757990 | FAM134C | 1,68E-02 | 0,809684809 |
| 3962054 | LLPH // LLPH | 1,70E-02 | 0,626521577 |
| 3092808 | NRG1 | 1,73E-02 | 0,719682288 |
| 3822849 | CLEC17A | 1,76E-02 | 0,812971563 |
| 3882984 | MAP1LC3A // MAP1LC3A | 1,79E-02 | 0,76994359 |
| 2974542 | TAAR5 | 1,79E-02 | 0,815641032 |
| 2799509 | C5orf38 | 1,82E-02 | 0,812678841 |
| 2623611 | GLYCTK // GLYCTK | 1,83E-02 | 0,828827298 |
| 3962000 | PMM1 // PMM1 | 1,83E-02 | 0,785948963 |
| 3339406 | FOLR1 | 1,84E-02 | 0,723658448 |
| 2427791 | DENND2D | 1,86E-02 | 0,833311873 |
| **2400027** | **PLA2G2A** | **1,89E-02** | **0,824136499** |
| 3718191 | CCL8 | 1,90E-02 | 0,616390092 |
| 2901787 | --- | 1,92E-02 | 0,625238652 |
| 2375784 | LINC00260 | 2,00E-02 | 0,761572572 |
| 3356417 | C11orf44 | 2,03E-02 | 0,829012003 |
| 3402978 | --- | 2,08E-02 | 0,477039101 |
| 3859703 | FAM187B | 2,13E-02 | 0,734373575 |
| 2335160 | FOXE3 | 2,14E-02 | 0,826131043 |
| 2339109 | MGC34796 | 2,15E-02 | 0,688676285 |
| 2704894 | PHC3 | 2,17E-02 | 0,688440001 |
| 3306523 | --- | 2,20E-02 | 0,522257901 |
| 3688178 | ZNF668 | 2,24E-02 | 0,789504636 |
| 2383747 | --- | 2,30E-02 | 0,717850229 |
| 3028911 | C7orf34 | 2,33E-02 | 0,786930852 |
| 2527672 | PNKD | 2,35E-02 | 0,823181659 |
| 4019599 | RNF113A | 2,36E-02 | 0,735197378 |
| 3557756 | NRL // NRL | 2,36E-02 | 0,767309036 |
| 3799167 | MPPE1 | 2,36E-02 | 0,636572091 |
| 3957666 | SMTN // SMTN | 2,43E-02 | 0,805374578 |
| 3380996 | ANAPC15 | 2,48E-02 | 0,630044368 |
| 3699777 | --- | 2,55E-02 | 0,688829788 |
| 4031692 | PRY | 2,57E-02 | 0,663615493 |
| 3355315 | KIRREL3-AS3 | 2,57E-02 | 0,823089802 |
| 3023211 | ATP6V1F | 2,59E-02 | 0,786296659 |
| 2950656 | ZBTB22 | 2,60E-02 | 0,79584379 |
| 3470260 | --- | 2,60E-02 | 0,78146645 |
| 2562115 | LSM3 // LSM3 | 2,61E-02 | 0,654523785 |
| 2318811 | --- | 2,62E-02 | 0,774825437 |
| 3096460 | --- | 2,62E-02 | 0,479982057 |
| 3907190 | SLPI // SLPI | 2,62E-02 | 0,792502556 |
| 3643297 | STUB1 // STUB1 | 2,64E-02 | 0,79229343 |
| 3031800 | ASIC3 | 2,64E-02 | 0,799738061 |
| 2489545 | --- | 2,66E-02 | 0,80628372 |
| 3268895 | --- | 2,66E-02 | 0,797821862 |
| 2595252 | SUMO1 // SUMO1 | 2,69E-02 | 0,525404649 |
| 2406252 | --- | 2,70E-02 | 0,809548366 |
| 2844253 | --- | 2,76E-02 | 0,700043963 |
| 2328488 | --- | 2,81E-02 | 0,543170165 |
| 3735505 | AANAT // AANAT | 2,82E-02 | 0,720257704 |
| 2322389 | NECAP2 // NECAP2 | 2,83E-02 | 0,598292937 |
| 2435410 | S100A11 // S100A11 | 2,85E-02 | 0,758198829 |
| 2853885 | GDNF | 2,86E-02 | 0,831204532 |
| 2442397 | TADA1 | 2,87E-02 | 0,828542279 |
| 3005266 | VKORC1L1 | 2,87E-02 | 0,561906997 |
| 3995975 | SSR4 | 2,87E-02 | 0,609355567 |
| 2648873 | GMPS | 2,90E-02 | 0,806866069 |
| 2390089 | OR2W5 | 2,93E-02 | 0,78834575 |
| 3217167 | CORO2A | 2,93E-02 | 0,803778089 |
| 3809820 | --- | 2,95E-02 | 0,681045936 |
| 3078435 | PDIA4 | 2,97E-02 | 0,566582049 |
| 3348748 | C11orf1 | 2,98E-02 | 0,609675131 |
| 3872849 | ZNF497 | 2,98E-02 | 0,815712532 |
| 3816143 | ADAT3 | 3,01E-02 | 0,813713728 |
| 2406254 | --- | 3,07E-02 | 0,617940348 |
| 3819719 | --- | 3,07E-02 | 0,301995118 |
| 3089853 | CHMP7 | 3,08E-02 | 0,801554659 |
| 3015216 | COPS6 | 3,09E-02 | 0,651483869 |
| **3598662** | **MAP2K1** | **3,09E-02** | **0,629414492** |
| 3825225 | C19orf60 // C19orf60 | 3,21E-02 | 0,819746644 |
| 2841304 | --- | 3,22E-02 | 0,728296436 |
| 3629494 | CLPX | 3,23E-02 | 0,681660691 |
| 3755316 | MLLT6 // MLLT6 | 3,23E-02 | 0,290126031 |
| 2383734 | --- | 3,23E-02 | 0,784736323 |
| 3846011 | SGTA | 3,24E-02 | 0,812416341 |
| 3453496 | --- | 3,27E-02 | 0,436149212 |
| 3914346 | NPBWR2 | 3,27E-02 | 0,689402456 |
| 3096466 | --- | 3,30E-02 | 0,67355091 |
| 2401609 | HMGCL | 3,30E-02 | 0,771896018 |
| 2812359 | NLN | 3,33E-02 | 0,784035003 |
| 3790529 | GRP | 3,36E-02 | 0,361120368 |
| 3841756 | KIR2DL2 // KIR2DL2 | 3,38E-02 | 0,656987643 |
| 3853178 | EPHX3 | 3,41E-02 | 0,611020666 |
| 3198289 | C9orf123 | 3,42E-02 | 0,429617962 |
| 2644014 | PCCB | 3,45E-02 | 0,635397617 |
| 3664785 | CKLF // CKLF | 3,46E-02 | 0,673197669 |
| 3432090 | ALDH2 | 3,48E-02 | 0,829380704 |
| 3743371 | ASGR1 | 3,52E-02 | 0,814707762 |
| 3038617 | NDUFA4 | 3,53E-02 | 0,514291098 |
| 3529799 | GMPR2 | 3,55E-02 | 0,694249961 |
| 3809673 | --- | 3,60E-02 | 0,645375843 |
| 2521239 | CCDC150 // CCDC150 | 3,64E-02 | 0,8157085 |
| 2571075 | ANAPC1 | 3,69E-02 | 0,595297213 |
| 2383789 | --- | 3,76E-02 | 0,591575234 |
| 2628682 | ARL6IP5 | 3,77E-02 | 0,808598053 |
| 2599371 | TMBIM1 | 3,78E-02 | 0,693488867 |
| 2728408 | REST | 3,81E-02 | 0,823608417 |
| 3224333 | OR1L8 | 3,86E-02 | 0,771345066 |
| 3874775 | PRND | 3,88E-02 | 0,679133156 |
| 3456260 | ATF7 // ATF7 | 3,90E-02 | 0,764982674 |
| 2333907 | RNF220 | 3,93E-02 | 0,770681902 |
| 3572263 | ACYP1 // ACYP1 | 3,95E-02 | 0,56365911 |
| 3764872 | CLTC // CLTC | 3,98E-02 | 0,660254987 |
| 3150844 | SNTB1 | 3,98E-02 | 0,811260005 |
| 3069955 | TSPAN12 | 4,00E-02 | 0,761789909 |
| 3430086 | TCP11L2 | 4,00E-02 | 0,462639174 |
| 3676262 | MSRB1 | 4,05E-02 | 0,817486131 |
| 3261053 | --- | 4,06E-02 | 0,808227952 |
| 2336706 | CPT2 | 4,13E-02 | 0,788552156 |
| 3275422 | --- | 4,14E-02 | 0,763783326 |
| 3674840 | POLR3K // POLR3K | 4,14E-02 | 0,349447666 |
| 3374793 | OR10V1 | 4,15E-02 | 0,819944932 |
| 2923939 | SMPDL3A | 4,17E-02 | 0,752950262 |
| 2716246 | FLJ35424 | 4,18E-02 | 0,822775393 |
| 2669157 | EPM2AIP1 | 4,19E-02 | 0,801438164 |
| 3830166 | FXYD3 // FXYD3 | 4,20E-02 | 0,700327123 |
| 3427014 | SNRPF | 4,23E-02 | 0,645148113 |
| 2757944 | TNIP2 | 4,24E-02 | 0,796602268 |
| 2820394 | NR2F1 | 4,25E-02 | 0,826841582 |
| 3861617 | HNRNPL | 4,26E-02 | 0,529935155 |
| 3133479 | RNF170 | 4,30E-02 | 0,635567297 |
| 3819739 | --- | 4,31E-02 | 0,781746294 |
| 3231121 | WDR85 | 4,33E-02 | 0,822496146 |
| 2430994 | ZNF697 | 4,33E-02 | 0,807950421 |
| 3631964 | PKM | 4,37E-02 | 0,702964656 |
| 3000953 | UPP1 | 4,37E-02 | 0,81439931 |
| 2536412 | --- | 4,38E-02 | 0,653724721 |
| 2555630 | CCT4 | 4,40E-02 | 0,705594389 |
| 2794584 | GPM6A | 4,47E-02 | 0,617574915 |
| 3337168 | GSTP1 | 4,54E-02 | 0,647418038 |
| 3096536 | --- | 4,56E-02 | 0,77476453 |
| 3556556 | OR6J1 | 4,67E-02 | 0,795334848 |
| 2934801 | MAP3K4 | 4,74E-02 | 0,756325368 |
| 2841348 | --- | 4,74E-02 | 0,770495442 |
| 3456081 | RARG | 4,78E-02 | 0,805694191 |
| 3570049 | ERH // ERH | 4,79E-02 | 0,746833643 |
| 2848265 | CMBL | 4,80E-02 | 0,542573482 |
| 3114649 | --- | 4,81E-02 | 0,80044335 |
| 2384375 | DUSP5P | 4,82E-02 | 0,723340157 |
| 3475782 | HCAR3 // HCAR3 | 4,83E-02 | 0,642795485 |
| 2905196 | RAB44 // RAB44 | 4,85E-02 | 0,830236401 |
| 3755820 | PGAP3 | 4,86E-02 | 0,776458435 |
| 2334279 | UROD // UROD | 4,90E-02 | 0,788197203 |
| 2662356 | TADA3 | 4,93E-02 | 0,832725246 |
| 3042603 | KIAA0087 | 4,96E-02 | 0,801570618 |
| 3639406 | FAM174B | 4,96E-02 | 0,677711434 |
| 3865223 | TRAPPC6A | 4,96E-02 | 0,588442284 |
| 3027943 | TAS2R3 | 4,98E-02 | 0,80974235 |

Table S3. List of upregulated genes in CCAM epithelium compared to control zones. These genes were selected using Ingenuity software with a filter p value ≤ 0.05 and a 1.2-fold alteration of probe expression.

| **Probes** | **Gene Symbol** | **pval** | **Mean Ratio CCAM vs Controls** |
| --- | --- | --- | --- |
| 2792166 | MARCH1 | 2,93E-04 | 1,543213767 |
| 2361584 | APOA1BP // APOA1BP | 5,75E-04 | 2,113043511 |
| 2482683 | RPL23AP32 // RPL23AP32 | 9,52E-04 | 3,327620231 |
| 3622386 | GATM | 9,96E-04 | 2,569734059 |
| 3775147 | FOXK2 // FOXK2 | 1,45E-03 | 3,238538766 |
| 3981735 | --- | 1,91E-03 | 8,444854594 |
| 3394080 | --- | 1,92E-03 | 1,320712695 |
| 3394068 | RPS25 | 1,92E-03 | 1,320712695 |
| 3746845 | TRIM16 | 1,92E-03 | 1,698806635 |
| 3180717 | ERCC6L2 | 1,94E-03 | 2,565713702 |
| 3722323 | --- | 2,36E-03 | 1,555642672 |
| 2841462 | --- | 2,42E-03 | 31,64391137 |
| 3544141 | ISCA2 | 2,60E-03 | 4,031753787 |
| 3833040 | SUPT5H | 3,29E-03 | 1,260312877 |
| 3275258 | --- | 3,39E-03 | 6,169739207 |
| 3984840 | ARMCX4 | 3,45E-03 | 1,588712698 |
| 3757664 | RAB5C | 3,60E-03 | 1,960494122 |
| 3110272 | FZD6 | 3,62E-03 | 1,9158501 |
| 3112543 | UTP23 | 3,66E-03 | 2,18527715 |
| 3258477 | PLCE1 | 3,66E-03 | 2,015958245 |
| 2939782 | --- | 4,48E-03 | 11,04539883 |
| 3903708 | TRPC4AP | 4,56E-03 | 1,656597477 |
| 3928538 | KRTAP19-5 | 5,42E-03 | 1,451148315 |
| 3957589 | MORC2 | 6,05E-03 | 1,310097548 |
| 3819661 | --- | 6,81E-03 | 3,354579993 |
| 2440018 | DCAF8 | 7,33E-03 | 1,2765036 |
| 3335842 | --- | 8,04E-03 | 2,398076806 |
| 2594627 | FAM126B // FAM126B | 8,04E-03 | 2,320217711 |
| 2609560 | THUMPD3 | 9,78E-03 | 2,038562557 |
| 3958269 | --- | 1,00E-02 | 3,212406905 |
| 2536334 | --- | 1,04E-02 | 2,253634693 |
| 3067080 | COG5 | 1,06E-02 | 1,582373962 |
| 2904702 | --- | 1,09E-02 | 2,458784488 |
| 3275326 | --- | 1,16E-02 | 1,20871831 |
| 3238962 | KIAA1217 | 1,21E-02 | 1,441820772 |
| 3851267 | ZNF625 // ZNF625 | 1,21E-02 | 1,498431315 |
| 3387413 | FAM76B | 1,23E-02 | 1,695366542 |
| 4022106 | MBNL3 | 1,23E-02 | 1,717480104 |
| 2899437 | BTN2A1 | 1,26E-02 | 1,423117368 |
| 3020222 | --- | 1,27E-02 | 6,021005669 |
| 3919834 | CBR3 | 1,28E-02 | 1,749591156 |
| 3725852 | --- | 1,34E-02 | 1,3029564 |
| 3729528 | PPM1D | 1,34E-02 | 1,4003763 |
| 3680524 | ZC3H7A | 1,37E-02 | 1,495181987 |
| 2891241 | DUSP22 | 1,37E-02 | 1,565472473 |
| 3542689 | PCNX | 1,37E-02 | 1,777350553 |
| 2844383 | --- | 1,40E-02 | 1,337607703 |
| 2962767 | PGM3 | 1,42E-02 | 2,597801564 |
| 2672467 | CCDC12 | 1,46E-02 | 1,323543076 |
| 3601051 | NEO1 | 1,48E-02 | 1,906548789 |
| 2730746 | SLC4A4 | 1,55E-02 | 1,945877321 |
| 3819565 | --- | 1,68E-02 | 10,68178202 |
| 3972093 | POLA1 | 1,69E-02 | 1,621002779 |
| 2370991 | DHX9 | 1,70E-02 | 1,449705173 |
| 3175494 | GCNT1 | 1,74E-02 | 1,601209125 |
| 3096520 | --- | 1,75E-02 | 1,288491183 |
| 3633522 | SNUPN | 1,79E-02 | 1,95107532 |
| 2872471 | DTWD2 | 1,79E-02 | 1,645782724 |
| 3416514 | --- | 1,80E-02 | 3,077826824 |
| 3015395 | PVRIG | 1,81E-02 | 1,674775174 |
| 2666103 | NKIRAS1 | 1,82E-02 | 2,011842811 |
| 3182019 | STX17 | 1,84E-02 | 3,468057288 |
| 2694931 | TMCC1 | 1,87E-02 | 1,51940038 |
| 3221135 | INIP | 1,91E-02 | 2,272490011 |
| 2603597 | PDE6D | 1,92E-02 | 1,691674334 |
| 3532935 | MIPOL1 | 1,95E-02 | 1,666172582 |
| 2777564 | FAM13A // FAM13A | 1,96E-02 | 2,08963181 |
| 3526151 | TUBGCP3 | 2,02E-02 | 1,467470464 |
| 2504883 | UGGT1 | 2,03E-02 | 1,674431162 |
| 3570083 | --- | 2,04E-02 | 6,947291653 |
| 3593770 | AP4E1 | 2,05E-02 | 1,680396729 |
| 2328541 | --- | 2,05E-02 | 1,203987914 |
| 3449068 | TMTC1 | 2,08E-02 | 1,202782562 |
| 2461531 | IRF2BP2 | 2,14E-02 | 1,30374794 |
| 3330798 | OR5L2 | 2,16E-02 | 1,463708811 |
| 2513554 | CSRNP3 // CSRNP3 | 2,22E-02 | 1,216947972 |
| 3264391 | VTI1A | 2,23E-02 | 2,739926238 |
| 3442054 | CHD4 | 2,23E-02 | 1,336899328 |
| 3474522 | --- | 2,24E-02 | 4,968401876 |
| 2451493 | CYB5R1 | 2,25E-02 | 1,494992974 |
| 3275256 | --- | 2,26E-02 | 3,55857024 |
| 2968281 | --- | 2,28E-02 | 11,03991807 |
| 2686727 | ZBTB11 | 2,34E-02 | 1,337764112 |
| 2840626 | --- | 2,37E-02 | 1,21169271 |
| 3384417 | --- | 2,39E-02 | 1,860006524 |
| 2831983 | --- | 2,40E-02 | 2,042285909 |
| 2318857 | --- | 2,42E-02 | 1,57959931 |
| 2844343 | --- | 2,50E-02 | 1,318962622 |
| 3216476 | ZNF510 | 2,51E-02 | 1,448824254 |
| 3453399 | --- | 2,57E-02 | 1,457374078 |
| 2992197 | SP4 | 2,58E-02 | 1,672819027 |
| 2627390 | --- | 2,60E-02 | 1,383618722 |
| 3390067 | NPAT | 2,60E-02 | 1,942088849 |
| 3147020 | ZNF706 | 2,62E-02 | 2,931266708 |
| 3837836 | CYTH2 | 2,64E-02 | 1,536411703 |
| 3749650 | --- | 2,64E-02 | 1,302181677 |
| 3625539 | NEDD4 | 2,79E-02 | 1,667966607 |
| **2380590** | **TGFB2** | **2,79E-02** | **1,968789784** |
| 3269587 | C10orf137 | 2,82E-02 | 1,78097658 |
| 3345107 | ANKRD49 | 2,85E-02 | 2,908448053 |
| 3749732 | --- | 2,87E-02 | 1,317483838 |
| 3627363 | NARG2 | 2,89E-02 | 1,833449945 |
| 3453388 | --- | 2,92E-02 | 3,251216539 |
| 3228621 | SURF6 | 2,92E-02 | 1,244153952 |
| 3335803 | --- | 2,96E-02 | 1,36366408 |
| 3749534 | --- | 3,01E-02 | 2,608991093 |
| 3212366 | --- | 3,01E-02 | 8,584970414 |
| 3556102 | --- | 3,06E-02 | 2,334439642 |
| 3253444 | --- | 3,07E-02 | 1,364890476 |
| 3699781 | --- | 3,10E-02 | 1,715816197 |
| 2828115 | LYRM7 // LYRM7 | 3,17E-02 | 3,67431266 |
| 2629782 | EBLN2 | 3,18E-02 | 1,887021434 |
| 3725801 | --- | 3,29E-02 | 7,392696765 |
| 2755053 | CYP4V2 | 3,30E-02 | 1,393160332 |
| 3621160 | ZSCAN29 | 3,37E-02 | 1,473418874 |
| 2674290 | --- | 3,41E-02 | 2,005303059 |
| 3463056 | CSRP2 // CSRP2 | 3,43E-02 | 1,48156713 |
| 4009315 | HUWE1 | 3,44E-02 | 1,257286272 |
| 3322070 | --- | 3,48E-02 | 1,38851412 |
| 2489258 | INO80B // INO80B | 3,50E-02 | 1,232418933 |
| **3181728** | **TGFBR1** | **3,54E-02** | **2,674161293** |
| 2359817 | INTS3 | 3,56E-02 | 1,334358421 |
| 3790259 | MALT1 | 3,59E-02 | 1,409583642 |
| 3257559 | RPP30 | 3,61E-02 | 2,643950812 |
| 3107151 | FAM92A1 | 3,62E-02 | 2,737444304 |
| 3872380 | ZNF154 | 3,66E-02 | 1,395443063 |
| 3591909 | CTDSPL2 | 3,69E-02 | 2,558664932 |
| 3015178 | --- | 3,75E-02 | 1,396924548 |
| 3015941 | SRRT | 3,77E-02 | 1,254762268 |
| 3474968 | --- | 3,77E-02 | 3,760812526 |
| 3191695 | EXOSC2 | 3,81E-02 | 2,650789729 |
| 2477073 | CRIM1 | 3,82E-02 | 1,364613566 |
| 2437753 | KIAA0907 | 3,82E-02 | 2,015677237 |
| 3058209 | MAGI2 | 3,84E-02 | 1,238875103 |
| 3191147 | TOR1B | 3,88E-02 | 1,358023774 |
| 3372253 | CELF1 | 3,89E-02 | 1,448709069 |
| 2777487 | FAM13A | 3,89E-02 | 1,699622706 |
| 4021508 | ZNF280C | 3,94E-02 | 1,411658996 |
| 3375951 | GANAB | 3,95E-02 | 1,359528493 |
| 2978050 | SHPRH | 3,96E-02 | 1,50007943 |
| 2575134 | POLR2D | 3,98E-02 | 1,489531647 |
| 4009062 | KDM5C | 4,02E-02 | 1,208587609 |
| 3360142 | TRIM21 | 4,03E-02 | 1,83954254 |
| 3645816 | ZNF75A | 4,08E-02 | 2,139188948 |
| 3521174 | ABCC4 | 4,08E-02 | 1,631189796 |
| 3473083 | MED13L | 4,11E-02 | 1,475909549 |
| 3420151 | MSRB3 | 4,13E-02 | 1,816851322 |
| 3427767 | TMPO | 4,16E-02 | 1,535216259 |
| 3551744 | --- | 4,17E-02 | 1,551949664 |
| 3142217 | PAG1 | 4,18E-02 | 1,545647729 |
| **3731826** | **PRKCA** | **4,19E-02** | **1,262041695** |
| 2934521 | SLC22A3 | 4,22E-02 | 1,4258347 |
| 2989141 | C7orf26 | 4,23E-02 | 1,252095749 |
| 3408505 | LRMP | 4,25E-02 | 1,469502808 |
| 3121023 | C8orf33 | 4,26E-02 | 1,239662416 |
| 3474521 | --- | 4,27E-02 | 3,743869919 |
| 3319137 | PPFIBP2 | 4,29E-02 | 1,854209349 |
| 3335799 | --- | 4,33E-02 | 1,835475683 |
| 2939469 | PXDC1 | 4,36E-02 | 1,363228396 |
| 2407439 | SF3A3 | 4,37E-02 | 1,818604215 |
| 3570073 | --- | 4,38E-02 | 6,807921858 |
| 3300350 | IDE | 4,41E-02 | 1,598302287 |
| 3599280 | SKOR1 | 4,45E-02 | 1,379963752 |
| 3517251 | DACH1 | 4,47E-02 | 1,292699874 |
| 3732373 | NOL11 | 4,49E-02 | 2,369102507 |
| 3703520 | C16orf95 // C16orf95 | 4,49E-02 | 1,349600492 |
| 2890148 | HNRNPH1 | 4,51E-02 | 1,235656049 |
| 3687475 | GDPD3 // GDPD3 | 4,52E-02 | 1,298445217 |
| 2896484 | MYLIP | 4,56E-02 | 1,766157209 |
| 3364306 | SOX6 | 4,56E-02 | 1,232650671 |
| 2635641 | PVRL3 | 4,58E-02 | 2,170256439 |
| 2585701 | STK39 | 4,59E-02 | 1,814196851 |
| 3559497 | STRN3 | 4,60E-02 | 1,610505358 |
| 2383891 | --- | 4,60E-02 | 1,512849465 |
| 3758209 | LOC388387 | 4,62E-02 | 1,244226295 |
| 3175274 | PCSK5 | 4,65E-02 | 1,524867242 |
| 2678116 | FAM116A | 4,68E-02 | 1,683797163 |
| 3108901 | VPS13B | 4,69E-02 | 1,32119238 |
| 2786578 | NDUFC1 | 4,70E-02 | 1,650446815 |
| 2617188 | ITGA9 | 4,71E-02 | 1,489661729 |
| 3275580 | --- | 4,71E-02 | 15,72451747 |
| 3765642 | INTS2 | 4,73E-02 | 1,637259945 |
| 2536474 | --- | 4,73E-02 | 5,263419207 |
| 3180342 | C9orf3 | 4,83E-02 | 1,277556395 |
| 3958267 | --- | 4,85E-02 | 3,345646314 |
| 2596763 | FZD5 // FZD5 | 4,88E-02 | 1,917225682 |
| 3191074 | NTMT1 | 4,92E-02 | 1,342961181 |
| 2907538 | PPP2R5D | 4,94E-02 | 1,390278268 |
| 3577940 | CLMN // CLMN | 4,95E-02 | 1,515892497 |
| 3685051 | USP31 | 4,97E-02 | 1,25962773 |

Table S4. Pathway analysis

| **Ingenuity Canonical Pathways** | **-log(p-value)** | **Ratio** | **z-score** | **Molecules** |
| --- | --- | --- | --- | --- |
| Wnt/Ca+ pathway | 2,42E+00 | 6,78E-02 | 1 | PLCD4,PRKCA,FZD6,PLCE1 |
| PPARα/RXRα Activation | 2,40E+00 | 3,80E-02 | -0,447 | PLCD4,PRKCA,NR2F1,TGFB2,PLCE1,TGFBR1,MAP2K1 |
| Synaptic Long Term Depression | 2,22E+00 | 4,00E-02 | -0,816 | PLCD4,PRKCA,PPP2R5D,PLCE1,MAP2K1,  PLA2G2A |
| Aldosterone Signaling in Epithelial Cells | 2,07E+00 | 3,70E-02 |  | NEDD4,PLCD4,PRKCA,PLCE1,MAP2K1,ASIC3 |
| Melatonin Signaling | 2,03E+00 | 5,26E-02 | 0 | PLCD4,PRKCA,PLCE1,MAP2K1 |
| Tight Junction Signaling | 2,01E+00 | 3,59E-02 |  | VTI1A,PPP2R5D,TGFB2,TGFBR1,MAGI2,PVRL3 |
| Wnt/β-catenin Signaling | 1,97E+00 | 3,53E-02 | -1 | SOX6,PPP2R5D,FZD6,TGFB2,TGFBR1,RARG |
| Regulation of IL-2 Expression in Activated and Anergic T Lymphocytes | 1,93E+00 | 4,94E-02 |  | MALT1,TGFB2,TGFBR1,MAP2K1 |
| p70S6K Signaling | 1,93E+00 | 4,00E-02 | -0,447 | PLCD4,PRKCA,PPP2R5D,PLCE1,MAP2K1 |
| Protein Kinase A Signaling | 1,92E+00 | 2,51E-02 |  | PLCD4,PRKCA,MPPE1,PDE6D,TGFB2,PLCE1,  TGFBR1,MAP2K1,DUSP22,ANAPC1 |
| Role of Oct4 in Mammalian Embryonic Stem Cell Pluripotency | 1,87E+00 | 6,52E-02 |  | REST,PHC3,NR2F1 |
| Sperm Motility | 1,80E+00 | 3,70E-02 | -0,447 | PLCD4,TWF1,PRKCA,PLCE1,PLA2G2A |
| Role of NFAT in Cardiac Hypertrophy | 1,79E+00 | 3,23E-02 | 0,816 | PLCD4,PRKCA,TGFB2,PLCE1,TGFBR1,MAP2K1 |
| Factors Promoting Cardiogenesis in Vertebrates | 1,75E+00 | 4,35E-02 |  | PRKCA,FZD6,TGFB2,TGFBR1 |
| Aryl Hydrocarbon Receptor Signaling | 1,67E+00 | 3,42E-02 | 1 | POLA1,NR2F1,TGFB2,RARG,GSTP1 |
| ErbB4 Signaling | 1,53E+00 | 4,84E-02 |  | PRKCA,NRG1,MAP2K1 |
| Antiproliferative Role of TOB in T Cell Signaling | 1,49E+00 | 7,69E-02 |  | TGFB2,TGFBR1 |
| Germ Cell-Sertoli Cell Junction Signaling | 1,49E+00 | 3,07E-02 |  | TGFB2,TGFBR1,MAP3K4,MAP2K1,PVRL3 |
| Phospholipases | 1,44E+00 | 4,48E-02 |  | PLCD4,PLCE1,PLA2G2A |
| Adenosine Nucleotides Degradation II | 1,43E+00 | 7,14E-02 |  | ADAT3,NT5C1A |
| 14-3-3-mediated Signaling | 1,39E+00 | 3,36E-02 |  | PLCD4,PRKCA,PLCE1,MAP2K1 |
| Cardiac Hypertrophy Signaling | 1,39E+00 | 2,59E-02 | 0 | PLCD4,TGFB2,PLCE1,TGFBR1,MAP3K4,  MAP2K1 |
| Role of MAPK Signaling in the Pathogenesis of Influenza | 1,38E+00 | 4,23E-02 |  | PRKCA,MAP2K1,PLA2G2A |
| Hepatic Cholestasis | 1,36E+00 | 2,82E-02 |  | PRKCA,SLCO3A1,TGFB2,MAP3K4,IL17B |
| CREB Signaling in Neurons | 1,34E+00 | 2,79E-02 | 0 | PLCD4,PRKCA,POLR2D,PLCE1,MAP2K1 |
| Caveolar-mediated Endocytosis Signaling | 1,33E+00 | 4,05E-02 |  | RAB5C,PRKCA,ITGA9 |
| RAR Activation | 1,31E+00 | 2,73E-02 |  | PRKCA,NR2F1,TGFB2,RARG,MAP2K1 |
| Synaptic Long Term Potentiation | 1,30E+00 | 3,15E-02 |  | PLCD4,PRKCA,PLCE1,MAP2K1 |
| P2Y Purigenic Receptor Signaling Pathway | 1,28E+00 | 3,10E-02 | 0 | PLCD4,PRKCA,PLCE1,MAP2K1 |
| Gustation Pathway | 1,28E+00 | 3,10E-02 |  | MPPE1,PDE6D,TAS2R3,ASIC3 |
| Leptin Signaling in Obesity | 1,28E+00 | 3,85E-02 |  | PLCD4,PLCE1,MAP2K1 |
| GPCR-Mediated Integration of Enteroendocrine Signaling Exemplified by an L Cell | 1,26E+00 | 3,80E-02 |  | PLCD4,GRP,PLCE1 |
| PI3K Signaling in B Lymphocytes | 1,24E+00 | 3,01E-02 |  | MALT1,PLCD4,PLCE1,MAP2K1 |
| VEGF Family Ligand-Receptor Interactions | 1,22E+00 | 3,66E-02 |  | PRKCA,MAP2K1,PLA2G2A |
| D-myo-inositol (1,4,5)-Trisphosphate Biosynthesis | 1,22E+00 | 5,41E-02 |  | PLCD4,PLCE1 |
| Purine Nucleotides Degradation II (Aerobic) | 1,22E+00 | 5,41E-02 |  | ADAT3,NT5C1A |
| IL-12 Signaling and Production in Macrophages | 1,19E+00 | 2,88E-02 |  | PRKCA,TGFB2,MAP2K1,ZNF668 |
| Human Embryonic Stem Cell Pluripotency | 1,19E+00 | 2,88E-02 |  | LEFTY2,FZD6,TGFB2,TGFBR1 |
| ErbB Signaling | 1,17E+00 | 3,49E-02 |  | PRKCA,NRG1,MAP2K1 |
| TGF-β Signaling | 1,16E+00 | 3,45E-02 |  | TGFB2,TGFBR1,MAP2K1 |
| UVA-Induced MAPK Signaling | 1,14E+00 | 3,37E-02 |  | PLCD4,PRKCA,PLCE1 |
| Mechanisms of Viral Exit from Host Cells | 1,14E+00 | 4,88E-02 |  | NEDD4,PRKCA |
| Thyroid Cancer Signaling | 1,14E+00 | 4,88E-02 |  | GDNF,MAP2K1 |
| Glutathione-mediated Detoxification | 1,14E+00 | 4,88E-02 |  | ANPEP,GSTP1 |
| tRNA Splicing | 1,12E+00 | 4,76E-02 |  | MPPE1,PDE6D |
| UVC-Induced MAPK Signaling | 1,12E+00 | 4,76E-02 |  | PRKCA,MAP2K1 |
| GPCR-Mediated Nutrient Sensing in Enteroendocrine Cells | 1,12E+00 | 3,30E-02 |  | PLCD4,PRKCA,PLCE1 |
| Epithelial Adherens Junction Signaling | 1,11E+00 | 2,70E-02 |  | TGFB2,TGFBR1,MAGI2,PVRL3 |
| Neuregulin Signaling | 1,09E+00 | 3,23E-02 |  | PRKCA,NRG1,MAP2K1 |
| Chronic Myeloid Leukemia Signaling | 1,09E+00 | 3,23E-02 |  | TGFB2,TGFBR1,MAP2K1 |
| D-myo-inositol-5-phosphate Metabolism | 1,09E+00 | 2,67E-02 |  | PLCD4,PPP2R5D,PLCE1,PPFIBP2 |
| Glioblastoma Multiforme Signaling | 1,08E+00 | 2,65E-02 | 0 | PLCD4,FZD6,PLCE1,MAP2K1 |
| Glycine Degradation (Creatine Biosynthesis) | 1,03E+00 | 1,11E-01 |  | GATM |
| T Cell Receptor Signaling | 1,00E+00 | 2,94E-02 |  | MALT1,PAG1,MAP2K1 |
| Antioxidant Action of Vitamin C | 9,94E-01 | 2,91E-02 |  | PLCD4,PLCE1,PLA2G2A |
| Embryonic Stem Cell Differentiation into Cardiac Lineages | 9,86E-01 | 1,00E-01 |  | SP4 |
| Neuropathic Pain Signaling In Dorsal Horn Neurons | 9,84E-01 | 2,88E-02 |  | PLCD4,PRKCA,PLCE1 |
| CD27 Signaling in Lymphocytes | 9,62E-01 | 3,85E-02 |  | MAP3K4,MAP2K1 |
| Gap Junction Signaling | 9,57E-01 | 2,38E-02 |  | PLCD4,PRKCA,PLCE1,MAP2K1 |
| Amyotrophic Lateral Sclerosis Signaling | 9,57E-01 | 2,80E-02 |  | RAB5C,GDNF,SSR4 |
| UVB-Induced MAPK Signaling | 9,48E-01 | 3,77E-02 |  | PRKCA,MAP2K1 |
| HGF Signaling | 9,48E-01 | 2,78E-02 |  | PRKCA,MAP3K4,MAP2K1 |
| Pancreatic Adenocarcinoma Signaling | 9,48E-01 | 2,78E-02 |  | TGFB2,TGFBR1,MAP2K1 |
| Pentose Phosphate Pathway (Oxidative Branch) | 9,47E-01 | 9,09E-02 |  | H6PD |
| Phenylethylamine Degradation I | 9,47E-01 | 9,09E-02 |  | ALDH2 |
| Methylmalonyl Pathway | 9,11E-01 | 8,33E-02 |  | PCCB |
| Dopamine-DARPP32 Feedback in cAMP Signaling | 9,10E-01 | 2,29E-02 |  | PLCD4,PRKCA,PPP2R5D,PLCE1 |
| ErbB2-ErbB3 Signaling | 8,96E-01 | 3,51E-02 |  | NRG1,MAP2K1 |
| Fc Epsilon RI Signaling | 8,88E-01 | 2,61E-02 |  | PRKCA,MAP2K1,PLA2G2A |
| NRF2-mediated Oxidative Stress Response | 8,78E-01 | 2,22E-02 |  | ABCC4,PRKCA,MAP2K1,GSTP1 |
| Thrombopoietin Signaling | 8,72E-01 | 3,39E-02 |  | PRKCA,MAP2K1 |
| p38 MAPK Signaling | 8,72E-01 | 2,56E-02 |  | TGFB2,TGFBR1,PLA2G2A |
| B Cell Receptor Signaling | 8,72E-01 | 2,21E-02 |  | MALT1,PAG1,MAP3K4,MAP2K1 |
| EGF Signaling | 8,60E-01 | 3,33E-02 |  | PRKCA,MAP2K1 |
| Regulation of the Epithelial-Mesenchymal Transition Pathway | 8,54E-01 | 2,17E-02 |  | FZD6,TGFB2,TGFBR1,MAP2K1 |
| DNA Double-Strand Break Repair by Homologous Recombination | 8,49E-01 | 7,14E-02 |  | POLA1 |
| HMGB1 Signaling | 8,49E-01 | 2,50E-02 |  | TGFB2,MAP2K1,IL17B |
| PTEN Signaling | 8,49E-01 | 2,50E-02 |  | TGFBR1,MAGI2,MAP2K1 |
| Endothelin-1 Signaling | 8,41E-01 | 2,15E-02 | 0 | PLCD4,PRKCA,PLCE1,PLA2G2A |
| Production of Nitric Oxide and Reactive Oxygen Species in Macrophages | 8,41E-01 | 2,15E-02 | -1 | PRKCA,PPP2R5D,MAP3K4,MAP2K1 |
| Sertoli Cell-Sertoli Cell Junction Signaling | 8,41E-01 | 2,15E-02 |  | MAP3K4,MAGI2,MAP2K1,PVRL3 |
| CCR3 Signaling in Eosinophils | 8,33E-01 | 2,46E-02 |  | PRKCA,MAP2K1,PLA2G2A |
| Cell Cycle: G1/S Checkpoint Regulation | 8,16E-01 | 3,12E-02 |  | TGFB2,NRG1 |
| ERK/MAPK Signaling | 8,12E-01 | 2,09E-02 | -1 | PRKCA,PPP2R5D,MAP2K1,PLA2G2A |
| Role of Pattern Recognition Receptors in Recognition of Bacteria and Viruses | 7,97E-01 | 2,36E-02 |  | PRKCA,TGFB2,IL17B |
| Estrogen Receptor Signaling | 7,97E-01 | 2,36E-02 |  | MED13L,POLR2D,MAP2K1 |
| Glycogen Degradation II | 7,96E-01 | 6,25E-02 |  | PGM3 |
| GDP-glucose Biosynthesis | 7,96E-01 | 6,25E-02 |  | PGM3 |
| Mitotic Roles of Polo-Like Kinase | 7,95E-01 | 3,03E-02 |  | PPP2R5D,ANAPC1 |
| Thrombin Signaling | 7,79E-01 | 2,03E-02 | 0 | PLCD4,PRKCA,PLCE1,MAP2K1 |
| UDP-N-acetyl-D-glucosamine Biosynthesis II | 7,72E-01 | 5,88E-02 |  | PGM3 |
| Adenine and Adenosine Salvage III | 7,72E-01 | 5,88E-02 |  | ADAT3 |
| 2-oxobutanoate Degradation I | 7,72E-01 | 5,88E-02 |  | PCCB |
| Erythropoietin Signaling | 7,55E-01 | 2,86E-02 |  | PRKCA,MAP2K1 |
| Xenobiotic Metabolism Signaling | 7,50E-01 | 1,82E-02 |  | PRKCA,PPP2R5D,MAP3K4,MAP2K1,GSTP1 |
| Ketogenesis | 7,49E-01 | 5,56E-02 |  | HMGCL |
| Glycogen Degradation III | 7,49E-01 | 5,56E-02 |  | PGM3 |
| IL-3 Signaling | 7,45E-01 | 2,82E-02 |  | PRKCA,MAP2K1 |
| Non-Small Cell Lung Cancer Signaling | 7,45E-01 | 2,82E-02 |  | PRKCA,MAP2K1 |
| GDNF Family Ligand-Receptor Interactions | 7,45E-01 | 2,82E-02 |  | GDNF,MAP2K1 |
| GNRH Signaling | 7,43E-01 | 2,22E-02 |  | PRKCA,MAP3K4,MAP2K1 |
| GADD45 Signaling | 7,28E-01 | 5,26E-02 |  | MAP3K4 |
| Fatty Acid α-oxidation | 7,28E-01 | 5,26E-02 |  | ALDH2 |
| LPS-stimulated MAPK Signaling | 7,27E-01 | 2,74E-02 |  | PRKCA,MAP2K1 |
| STAT3 Pathway | 7,18E-01 | 2,70E-02 |  | TGFBR1,MAP2K1 |
| Chemokine Signaling | 7,09E-01 | 2,67E-02 |  | PRKCA,MAP2K1 |
| DNA Methylation and Transcriptional Repression Signaling | 7,08E-01 | 5,00E-02 |  | CHD4 |
| nNOS Signaling in Skeletal Muscle Cells | 7,08E-01 | 5,00E-02 |  | SNTB1 |
| Glucose and Glucose-1-phosphate Degradation | 7,08E-01 | 5,00E-02 |  | PGM3 |
| Purine Ribonucleosides Degradation to Ribose-1-phosphate | 7,08E-01 | 5,00E-02 |  | ADAT3 |
| Cardiac β-adrenergic Signaling | 7,05E-01 | 2,13E-02 |  | MPPE1,PDE6D,PPP2R5D |
| Prolactin Signaling | 7,00E-01 | 2,63E-02 |  | PRKCA,MAP2K1 |
| HER-2 Signaling in Breast Cancer | 7,00E-01 | 2,63E-02 |  | PRKCA,NRG1 |
| Mitochondrial L-carnitine Shuttle Pathway | 6,89E-01 | 4,76E-02 |  | CPT2 |
| Superpathway of Inositol Phosphate Compounds | 6,87E-01 | 1,86E-02 |  | PLCD4,PPP2R5D,PLCE1,PPFIBP2 |
| Cyclins and Cell Cycle Regulation | 6,84E-01 | 2,56E-02 |  | PPP2R5D,TGFB2 |
| Relaxin Signaling | 6,81E-01 | 2,07E-02 |  | MPPE1,PDE6D,MAP2K1 |
| VDR/RXR Activation | 6,75E-01 | 2,53E-02 |  | PRKCA,TGFB2 |
| AMPK Signaling | 6,58E-01 | 2,01E-02 |  | PPM1D,PPP2R5D,CPT2 |
| Urate Biosynthesis/Inosine 5'-phosphate Degradation | 6,54E-01 | 4,35E-02 |  | NT5C1A |
| Pentose Phosphate Pathway | 6,54E-01 | 4,35E-02 |  | H6PD |
| Guanosine Nucleotides Degradation III | 6,54E-01 | 4,35E-02 |  | NT5C1A |
| Regulation of eIF4 and p70S6K Signaling | 6,53E-01 | 2,00E-02 |  | PPP2R5D,MAP2K1,RPS25 |
| PDGF Signaling | 6,44E-01 | 2,41E-02 |  | PRKCA,MAP2K1 |
| UDP-N-acetyl-D-galactosamine Biosynthesis II | 6,38E-01 | 4,17E-02 |  | PGM3 |
| Ceramide Signaling | 6,36E-01 | 2,38E-02 |  | PPP2R5D,MAP2K1 |
| Role of Macrophages, Fibroblasts and Endothelial Cells in Rheumatoid Arthritis | 6,29E-01 | 1,64E-02 |  | PLCD4,PRKCA,FZD6,PLCE1,MAP2K1 |
| Prostate Cancer Signaling | 6,21E-01 | 2,33E-02 |  | MAP2K1,GSTP1 |
| Leucine Degradation I | 6,07E-01 | 3,85E-02 |  | HMGCL |
| RANK Signaling in Osteoclasts | 6,00E-01 | 2,25E-02 |  | MAP3K4,MAP2K1 |
| FGF Signaling | 6,00E-01 | 2,25E-02 |  | PRKCA,MAP2K1 |
| Apoptosis Signaling | 6,00E-01 | 2,25E-02 |  | PRKCA,MAP2K1 |
| Huntington's Disease Signaling | 5,95E-01 | 1,69E-02 |  | REST,VTI1A,PRKCA,POLR2D |
| IL-15 Production | 5,93E-01 | 3,70E-02 |  | TWF1 |
| Histamine Degradation | 5,93E-01 | 3,70E-02 |  | ALDH2 |
| Tryptophan Degradation X (Mammalian, via Tryptamine) | 5,80E-01 | 3,57E-02 |  | ALDH2 |
| γ-glutamyl Cycle | 5,80E-01 | 3,57E-02 |  | ANPEP |
| Oxidative Ethanol Degradation III | 5,80E-01 | 3,57E-02 |  | ALDH2 |
| Colorectal Cancer Metastasis Signaling | 5,76E-01 | 1,66E-02 |  | FZD6,TGFB2,TGFBR1,MAP2K1 |
| Putrescine Degradation III | 5,67E-01 | 3,45E-02 |  | ALDH2 |
| Ethanol Degradation IV | 5,67E-01 | 3,45E-02 |  | ALDH2 |
| PPAR Signaling | 5,67E-01 | 2,13E-02 |  | NR2F1,MAP2K1 |
| Virus Entry via Endocytic Pathways | 5,60E-01 | 2,11E-02 |  | PRKCA,FOLR1 |
| Mouse Embryonic Stem Cell Pluripotency | 5,60E-01 | 2,11E-02 |  | FZD6,MAP2K1 |
| Phospholipase C Signaling | 5,53E-01 | 1,62E-02 | 0 | PRKCA,PLCE1,MAP2K1,PLA2G2A |
| α-Adrenergic Signaling | 5,48E-01 | 2,06E-02 |  | PRKCA,MAP2K1 |
| VEGF Signaling | 5,48E-01 | 2,06E-02 |  | PRKCA,MAP2K1 |
| 4-1BB Signaling in T Lymphocytes | 5,42E-01 | 3,23E-02 |  | MAP2K1 |
| Telomerase Signaling | 5,36E-01 | 2,02E-02 |  | PPP2R5D,MAP2K1 |
| IGF-1 Signaling | 5,36E-01 | 2,02E-02 |  | NEDD4,MAP2K1 |
| Phenylalanine Degradation IV (Mammalian, via Side Chain) | 5,31E-01 | 3,12E-02 |  | ALDH2 |
| Glioma Signaling | 5,30E-01 | 2,00E-02 |  | PRKCA,MAP2K1 |
| CDK5 Signaling | 5,12E-01 | 1,94E-02 |  | PPP2R5D,MAP2K1 |
| Oncostatin M Signaling | 5,09E-01 | 2,94E-02 |  | MAP2K1 |
| Cell Cycle Regulation by BTG Family Proteins | 4,98E-01 | 2,86E-02 |  | PPP2R5D |
| MIF-mediated Glucocorticoid Regulation | 4,98E-01 | 2,86E-02 |  | PLA2G2A |
| NAD Salvage Pathway II | 4,98E-01 | 2,86E-02 |  | NT5C1A |
| Nucleotide Excision Repair Pathway | 4,98E-01 | 2,86E-02 |  | POLR2D |
| Cholecystokinin/Gastrin-mediated Signaling | 4,96E-01 | 1,89E-02 |  | PRKCA,MAP2K1 |
| G-Protein Coupled Receptor Signaling | 4,93E-01 | 1,52E-02 |  | PRKCA,MPPE1,PDE6D,MAP2K1 |
| Glucocorticoid Receptor Signaling | 4,83E-01 | 1,50E-02 |  | POLR2D,TGFB2,TGFBR1,MAP2K1 |
| Role of NANOG in Mammalian Embryonic Stem Cell Pluripotency | 4,70E-01 | 1,80E-02 |  | FZD6,MAP2K1 |
| Dopamine Degradation | 4,69E-01 | 2,63E-02 |  | ALDH2 |
| Axonal Guidance Signaling | 4,67E-01 | 1,37E-02 |  | C9orf3,PLCD4,PRKCA,FZD6,PLCE1,MAP2K1 |
| Salvage Pathways of Pyrimidine Ribonucleotides | 4,65E-01 | 1,79E-02 |  | UPP1,MAP2K1 |
| Nitric Oxide Signaling in the Cardiovascular System | 4,65E-01 | 1,79E-02 |  | PRKCA,MAP2K1 |
| Inhibition of Angiogenesis by TSP1 | 4,60E-01 | 2,56E-02 |  | TGFBR1 |
| Sphingosine-1-phosphate Signaling | 4,60E-01 | 1,77E-02 |  | PLCD4,PLCE1 |
| NGF Signaling | 4,60E-01 | 1,77E-02 |  | MAP3K4,MAP2K1 |
| Androgen Signaling | 4,55E-01 | 1,75E-02 |  | PRKCA,POLR2D |
| mTOR Signaling | 4,54E-01 | 1,55E-02 |  | PRKCA,PPP2R5D,RPS25 |
| Transcriptional Regulatory Network in Embryonic Stem Cells | 4,51E-01 | 2,50E-02 |  | REST |
| Natural Killer Cell Signaling | 4,50E-01 | 1,74E-02 |  | PRKCA,MAP2K1 |
| fMLP Signaling in Neutrophils | 4,50E-01 | 1,74E-02 |  | PRKCA,MAP2K1 |
| Breast Cancer Regulation by Stathmin1 | 4,43E-01 | 1,52E-02 |  | PRKCA,PPP2R5D,MAP2K1 |
| TCA Cycle II (Eukaryotic) | 4,43E-01 | 2,44E-02 |  | SUCLG1 |
| Glycolysis I | 4,43E-01 | 2,44E-02 |  | PKM |
| Renin-Angiotensin Signaling | 4,36E-01 | 1,69E-02 |  | PRKCA,MAP2K1 |
| Purine Nucleotides De Novo Biosynthesis II | 4,35E-01 | 2,38E-02 |  | GMPS |
| Ethanol Degradation II | 4,27E-01 | 2,33E-02 |  | ALDH2 |
| Corticotropin Releasing Hormone Signaling | 4,22E-01 | 1,65E-02 |  | PRKCA,MAP2K1 |
| Molecular Mechanisms of Cancer | 4,21E-01 | 1,34E-02 |  | PRKCA,FZD6,TGFB2,TGFBR1,MAP2K1 |
| MIF Regulation of Innate Immunity | 4,19E-01 | 2,27E-02 |  | PLA2G2A |
| Melanoma Signaling | 4,19E-01 | 2,27E-02 |  | MAP2K1 |
| Role of IL-17F in Allergic Inflammatory Airway Diseases | 4,19E-01 | 2,27E-02 |  | MAP2K1 |
| PKCθ Signaling in T Lymphocytes | 4,18E-01 | 1,64E-02 |  | MALT1,MAP3K4 |
| CD28 Signaling in T Helper Cells | 4,14E-01 | 1,63E-02 |  | MALT1,MAP2K1 |
| Neuroprotective Role of THOP1 in Alzheimer's Disease | 4,04E-01 | 2,17E-02 |  | IDE |
| PI3K/AKT Signaling | 3,93E-01 | 1,56E-02 |  | PPP2R5D,MAP2K1 |
| Cell Cycle: G2/M DNA Damage Checkpoint Regulation | 3,83E-01 | 2,04E-02 |  | PPM1D |
| Assembly of RNA Polymerase II Complex | 3,76E-01 | 2,00E-02 |  | POLR2D |
| Ovarian Cancer Signaling | 3,73E-01 | 1,50E-02 |  | FZD6,MAP2K1 |
| D-myo-inositol (1,4,5,6)-Tetrakisphosphate Biosynthesis | 3,73E-01 | 1,50E-02 |  | PPP2R5D,PPFIBP2 |
| D-myo-inositol (3,4,5,6)-tetrakisphosphate Biosynthesis | 3,73E-01 | 1,50E-02 |  | PPP2R5D,PPFIBP2 |
| CNTF Signaling | 3,63E-01 | 1,92E-02 |  | MAP2K1 |
| nNOS Signaling in Neurons | 3,63E-01 | 1,92E-02 |  | PRKCA |
| Noradrenaline and Adrenaline Degradation | 3,63E-01 | 1,92E-02 |  | ALDH2 |
| cAMP-mediated signaling | 3,61E-01 | 1,35E-02 |  | MPPE1,PDE6D,MAP2K1 |
| LPS/IL-1 Mediated Inhibition of RXR Function | 3,58E-01 | 1,34E-02 |  | ABCC4,CPT2,GSTP1 |
| Type II Diabetes Mellitus Signaling | 3,54E-01 | 1,45E-02 |  | PRKCA,PKM |
| Endometrial Cancer Signaling | 3,51E-01 | 1,85E-02 |  | MAP2K1 |
| Insulin Receptor Signaling | 3,47E-01 | 1,43E-02 |  | MAP2K1,ASIC3 |
| Role of CHK Proteins in Cell Cycle Checkpoint Control | 3,46E-01 | 1,82E-02 |  | PPP2R5D |
| IL-2 Signaling | 3,46E-01 | 1,82E-02 |  | MAP2K1 |
| Cellular Effects of Sildenafil (Viagra) | 3,40E-01 | 1,41E-02 |  | PLCD4,PLCE1 |
| Role of IL-17A in Arthritis | 3,40E-01 | 1,79E-02 |  | MAP2K1 |
| Phototransduction Pathway | 3,13E-01 | 1,64E-02 |  | PDE6D |
| 3-phosphoinositide Degradation | 3,11E-01 | 1,32E-02 |  | PPP2R5D,PPFIBP2 |
| PCP pathway | 3,03E-01 | 1,59E-02 |  | FZD6 |
| GM-CSF Signaling | 3,03E-01 | 1,59E-02 |  | MAP2K1 |
| Gαq Signaling | 2,99E-01 | 1,29E-02 |  | PRKCA,MAP2K1 |
| CD40 Signaling | 2,94E-01 | 1,54E-02 |  | MAP2K1 |
| IL-17A Signaling in Airway Cells | 2,89E-01 | 1,52E-02 |  | MAP2K1 |
| Superpathway of Methionine Degradation | 2,89E-01 | 1,52E-02 |  | PCCB |
| IL-15 Signaling | 2,85E-01 | 1,49E-02 |  | MAP2K1 |
| Antiproliferative Role of Somatostatin Receptor 2 | 2,85E-01 | 1,49E-02 |  | MAP2K1 |
| Retinoic acid Mediated Apoptosis Signaling | 2,85E-01 | 1,49E-02 |  | RARG |
| CXCR4 Signaling | 2,84E-01 | 1,25E-02 |  | PRKCA,MAP2K1 |
| Calcium-induced T Lymphocyte Apoptosis | 2,81E-01 | 1,47E-02 |  | PRKCA |
| Role of PI3K/AKT Signaling in the Pathogenesis of Influenza | 2,81E-01 | 1,47E-02 |  | MAP2K1 |
| Remodeling of Epithelial Adherens Junctions | 2,81E-01 | 1,47E-02 |  | RAB5C |
| Macropinocytosis Signaling | 2,81E-01 | 1,47E-02 |  | PRKCA |
| Agrin Interactions at Neuromuscular Junction | 2,72E-01 | 1,43E-02 |  | NRG1 |
| Protein Ubiquitination Pathway | 2,72E-01 | 1,16E-02 |  | NEDD4,USP31,ANAPC1 |
| PEDF Signaling | 2,68E-01 | 1,41E-02 |  | GDNF |
| IL-17 Signaling | 2,64E-01 | 1,39E-02 |  | MAP2K1 |
| T Helper Cell Differentiation | 2,64E-01 | 1,39E-02 |  | TGFBR1 |
| JAK/Stat Signaling | 2,64E-01 | 1,39E-02 |  | MAP2K1 |
| Neurotrophin/TRK Signaling | 2,64E-01 | 1,39E-02 |  | MAP2K1 |
| 3-phosphoinositide Biosynthesis | 2,63E-01 | 1,19E-02 |  | PPP2R5D,PPFIBP2 |
| Growth Hormone Signaling | 2,60E-01 | 1,37E-02 |  | PRKCA |
| Renal Cell Carcinoma Signaling | 2,60E-01 | 1,37E-02 |  | MAP2K1 |
| Basal Cell Carcinoma Signaling | 2,60E-01 | 1,37E-02 |  | FZD6 |
| NF-κB Activation by Viruses | 2,56E-01 | 1,35E-02 |  | PRKCA |
| CCR5 Signaling in Macrophages | 2,56E-01 | 1,35E-02 |  | PRKCA |
| FLT3 Signaling in Hematopoietic Progenitor Cells | 2,56E-01 | 1,35E-02 |  | MAP2K1 |
| Pyridoxal 5'-phosphate Salvage Pathway | 2,56E-01 | 1,35E-02 |  | MAP2K1 |
| NF-κB Signaling | 2,51E-01 | 1,16E-02 |  | MALT1,TGFBR1 |
| Acute Myeloid Leukemia Signaling | 2,45E-01 | 1,30E-02 |  | MAP2K1 |
| Serotonin Degradation | 2,45E-01 | 1,30E-02 |  | ALDH2 |
| BMP signaling pathway | 2,45E-01 | 1,30E-02 |  | MAP2K1 |
| Role of Wnt/GSK-3β Signaling in the Pathogenesis of Influenza | 2,38E-01 | 1,27E-02 |  | FZD6 |
| Dendritic Cell Maturation | 2,36E-01 | 1,12E-02 |  | PLCD4,PLCE1 |
| Eicosanoid Signaling | 2,31E-01 | 1,23E-02 |  | PLA2G2A |
| Melanocyte Development and Pigmentation Signaling | 2,15E-01 | 1,16E-02 |  | MAP2K1 |
| Bladder Cancer Signaling | 2,12E-01 | 1,15E-02 |  | MAP2K1 |
| HIPPO signaling | 2,12E-01 | 1,15E-02 |  | PPP2R5D |
| CTLA4 Signaling in Cytotoxic T Lymphocytes | 2,09E-01 | 1,14E-02 |  | PPP2R5D |
| FAK Signaling | 2,04E-01 | 1,11E-02 |  | MAP2K1 |
| PAK Signaling | 2,01E-01 | 1,10E-02 |  | MAP2K1 |
| Dopamine Receptor Signaling | 1,98E-01 | 1,09E-02 |  | PPP2R5D |

Table S5. Upstream analysis

| Upstream Regulator | Predicted Activation State | Activation z-score | p-value of overlap | Target molecules in dataset |
| --- | --- | --- | --- | --- |
| NR2E1 |  |  | 1,59E-03 | PLCE1,TGFB2,TGFBR1 |
| KLF6 |  |  | 7,82E-03 | NR2F1,RARG,TGFBR1 |
| HOXB9 |  |  | 1,42E-02 | NRG1,TGFB2 |
| HEXIM1 |  |  | 2,66E-02 | HNRNPH1,TGFB2 |
| HIF1A |  |  | 2,85E-02 | FAM13A,HMGCL,HUWE1,KIAA1217,PKM,PRKCA,  TGFB2,TRIM21 |
| FGF1 |  |  | 2,86E-02 | DCAF8,GDNF,GSTP1,TGFB2 |
| mir-25 |  |  | 3,07E-02 | MYLIP,TGFBR1 |
| WNT11 |  |  | 3,73E-02 | GDNF,TGFB2 |
| FSH |  | 0,469 | 4,56E-02 | ARL6IP5,MAP2K1,NEO1,PKM,RAB5C,TGFB2,TGFBR1,UPP1 |
| MSX2 |  |  | 4,94E-02 | FOXE3,TGFB2 |
| ADCYAP1 | Inhibited | -2 | 2,19E-01 | GRP,SELT,TGFB2,TMPO |
| CEBPA |  | -1,964 | 2,43E-01 | ANPEP,ARL6IP5,GSTP1,MALT1,SMPDL3A,TGFB2 |
| IFNG | Inhibited | -2,613 | 2,85E-01 | ARL6IP5,CCL8,CTSV,DHX9,GDNF,GNL1,H6PD,IDE,MAP2K1,MORC2,PLA2G2A,PRKCA,TGFB2,TGFBR1,  TRIM21 |
| CREB1 | Inhibited | -2,449 | 2,94E-01 | CRIM1,NEO1,NRSN1,PVRL3,SSR4,TGFBR1,UPP1 |
| IL6 | Inhibited | -2 | 1,00E+00 | ANPEP,HFE2,LEFTY2,MAP2K1,PLA2G2A |
